# Comparative Dissemination of Aerosol and Splatter Using Suction Device during Ultrasonic Scaling: A Pilot Study

**DOI:** 10.3390/dj10080142

**Published:** 2022-08-01

**Authors:** Nutthawadee Engsomboon, Praewpat Pachimsawat, Bhornsawan Thanathornwong

**Affiliations:** 1 Faculty of Dentistry, Srinakharinwirot University, Bangkok 10110, Thailand; nutthawadee@g.swu.ac.th; 2 Faculty of Dentistry, Mahidol University, Bangkok 10400, Thailand; praewpat.pac@mahidol.ac.th

**Keywords:** aerosols, dental equipment, dental scaling, suction, algorithms

## Abstract

Objective: This study compared the aerosol and splatter diameter and count numbers produced by a dental mouth prop with a suction holder device and a saliva ejector during ultrasonic scaling in a clinical setting. Methodology: Fluorescein dye was placed in the dental equipment irrigation reservoirs with a mannequin, and an ultrasonic scaler was employed. The procedures were performed three times per device. The upper and bottom board papers were placed on the laboratory platform. All processes used an ultrasonic scaler to generate aerosol and splatter. A dental mouth prop with a suction holder and a saliva ejector were also tested. Photographic analysis was used to examine the fluorescein samples, followed by image processing in Python and assessment of the diameter and count number. For device comparison, statistics were used with an independent *t*-test. Result: When using the dental mouth prop with a suction holder, the scaler produced aerosol particles that were maintained on the upper board paper (mean ± SD: 1080 ± 662 µm) compared to on the bottom board paper (1230 ± 1020 µm). When the saliva ejector was used, it was found that the diameter of the aerosol on the upper board paper was 900 ± 580 µm, and the diameter on the bottom board paper was 1000 ± 756 µm. Conclusion: There was a significant difference in the aerosol and splatter particle diameter and count number between the dental mouth prop with a suction holder and saliva ejector (*p* < 0.05). Furthermore, the results revealed that there was a statistically significant difference between the two groups on the upper and bottom board papers.

## 1. Introduction

In the dental clinic, undergraduate dental students still lack adequate operating experience and skill. Furthermore, the faculty of dentistry does not provide each dental student with a dental assistant. Moreover, dental work is a difficult process that necessitates a good view of the operation field, and the procedures are frequently associated with patient saliva, dispersed aerosol, and splatter [1,2,3]. Moreover, aerosol and splatter are carriers of infection from the blood and saliva [4]. The difference between aerosol and splatter is the size of the particle. Aerosols and splatters are defined by the World Health Organization [5] as having a diameter ≤50 and ≥50 µm, respectively. Due to the airborne spread of splattered fluid, droplets, and aerosols, the risk of transmitting potentially pathogenic bacteria (such as *Legionella pneumophila* or *Pseudomomas aeruginosa*) and both oral and respiratory viruses, including human influenza viruses and SARS-CoV-2, is a major concern in dental practice. Aerosols with smaller particle sizes have a higher risk of transmitting respiratory infections [6,7,8]. Especially during the scaling procedure, the use of an ultrasonic scaler is the greatest producer of contaminated aerosol and splatter [9]. 

However, the saliva ejector is the only low-velocity air evacuation equipment available for scaling and polishing, restorative, and prosthodontics procedures in the undergraduate dental student clinic. Traditionally, undergraduate dental students in years 4, 5, and 6 provide treatment without the help of a dental assistant. They require a saliva suction hose to make patients feel more at ease and can also work [10]. In the dental clinic, the assistant helps by holding the saliva hose; however, in the undergraduate dental student clinic, dental students hang the suction hose in the patient’s mouth or use their non-dominant hand to hold the suction hose. The dominant hand, which is skilled at manipulating tools, should be used for dental procedures. As mentioned above, it is evident that this situation is a cause of stress for both patients and dental students. The dental mouth prop with a suction holding device is designed to attach to a low-volume suction hose. It can provide suction in both the maxillary and mandibular quadrants and be used on the right and left side to alleviate dental students’ concerns during the procedure. Other advantages of the device include prevention of oro-muscular fatigue during extended visits and accidental patient closure of the mouth, which could result in trauma, and reduction of moisture contamination of the treated area. It is routinely used in dentistry to improve the quality of dental treatment because it can provide good access in the operation area, decrease the moisture in the patient’s mouth, and increase the patient’s comfort and safety. However, the amount of aerosol and splatter spread by dental equipment is undetermined. Finally, researchers expect that the use of this device in the dental clinic will improve dental students’ performance visually and take less time to complete each treatment, since they will not have to seek a saliva hose. Furthermore, the good impressions of the patients from dental students will be affected.

Therefore, in this study, a simulated clinical study was conducted to compare the dental mouth prop with a suction holder device and a saliva ejector and assess the aerosol and splatter diameter and count number during ultrasonic scaling. As a secondary purpose, the researchers also aimed to develop a new approach to measure aerosol and splatter spread.

## 2. Materials and Methods

### 2.1. Laboratory Platform Setup

**Figure 1:**
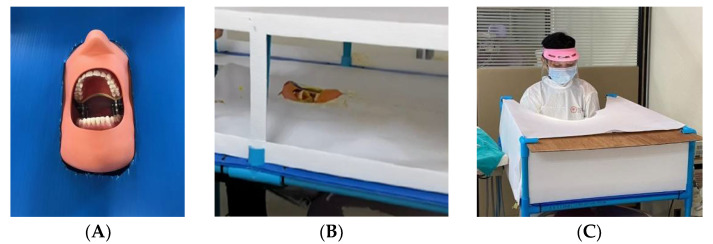
(**A**) A typodont manikin head setting. (**B**) The laboratory platform before it was covered by papers. (**C**) The operator worked in the laboratory platform set-up stand.

In a dental clinic, the researchers constructed a laboratory platform to compare the aerosol and splatter pattern obtained during a simulated scaling procedure. A 26 × 34 × 10-inch PVC pipe platform was built to enclose the manikin head as it was reclined into a usual position for scaling so that the maxillary dental occlusal plane was perpendicular to the floor [11]. A typodont manikin head (Nissin Dental, Kyoto, Japan) was placed in a dental chair’s headrest position. Then, a typodont (D16FE500H (GSF)-MF 28 teeth soft gingivae type, Nissin, Kyoto, Japan) was inserted into the maxillary and mandibular positions of the manikin head. The PVC pipe platform was covered with a blue polypropylene (PP) corrugated board that was trimmed to fit. The laboratory platform was set up with stands for support at a height of 26 inches around the manikin at the 12 o’clock position. In order to prevent airflow currents affecting the aerosol and splatter pattern, papers were placed around the laboratory platform (Figure 1). 

### 2.2. Experimental Design

A repeated-measures laboratory study was conducted in the dental clinic of the faculty of dentistry at Srinakharinwirot University. Six trials were carried out by a single operator using standard scaling techniques and operator positions were incorporated [5,6,7]. The operator (B.T) was a licensed dentist with over 15 years of expertise in the field. Aerosol and splatter were produced utilizing a Thai dental product, the Superson Mark III electromagnetic 25,000 cycles per second, manually tuned ultrasonic sealer, fitted with a P-10 series tip insert. Following the directions of the manufacturer, prior to the onset of scaling, the water lines were flushed. For all trials, the power and water flow indicator knobs on the ultrasonic instrument were set to medium. The operator performed timed simulated scaling on the facial and lingual portions of maxillary teeth assigned tooth numbers 17 to 25 of a Dentoform^®^ (Kyoto, Japan) model covered with a Nissin Mask M (w/Single Drain).

**Figure 2:**
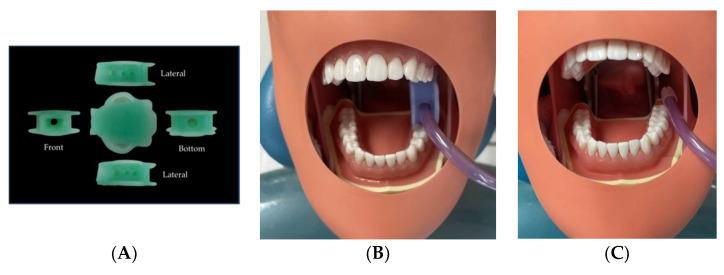
(**A**) The dental mouth prop with the suction holder device (Thai Patent No: 19329, Thai FDA No: 64-1-3-2-0000523). (**B**) The location at which the dental mouth prop with a suction holder attached to the saliva ejector was placed in the typodont manikin head. (**C**) The location at which the saliva ejector was placed.

Simulated upper arch scaling was used in the experiment, along with a low-volume evacuation system with a saliva ejector. Another experimental condition included the same simulated situation but with the addition of a dental mouth prop with a suction holder and saliva ejector. The dental mouth prop with a suction holder attached to the saliva ejector provided simultaneous suction to the maxillary and mandibular quadrants. The device also assisted in mouth opening, as a porous structure that allows fluid evacuation to be easily managed, and hands-free suction (Figure 2).

Sodium fluorescein (Himedia, India) was added to the coolant water to give a final concentration of 10 mg/mL [6,11]. The water spray aerosolized and scattered away from the ultrasonic dental scaler during the simulated scaling procedure; the resulting aerosol and splatter that dropped surrounded the typodont mouth and landed on the paper-covered platform. The procedures were carried out for 5 min. After each test, 2 board papers were allowed to thoroughly dry for 10 min and removed. Then, we allocated a number of the aerosol and splatter particles to each piece of covered paper. Then, the amount of aerosol and spatter particles on each piece of paper was measured.

### 2.3. Effects of Dental Suction

In a laboratory platform setup, researchers covered the air inlet vent to the operatory so that no airflow currents were present, as this could have affected the aerosol and splatter pattern. The suction flow rate was controlled at a standard low-volume evacuation level [12]. After, the saliva ejector and the dental mouth prop with a suction holder and saliva ejector device were placed in a 2-liter graduated cylinder filled with 2000 mL of water and their suction rates were tested. In 50 s, the saliva ejector cleared all of the water in the cylinder, equal to a rate of 40 mL/s. All of the water in the cylinder was cleared at the same time with a dental mouth prop with a suction holder and a saliva ejector device.

### 2.4. Image Processing

**Figure 3:**
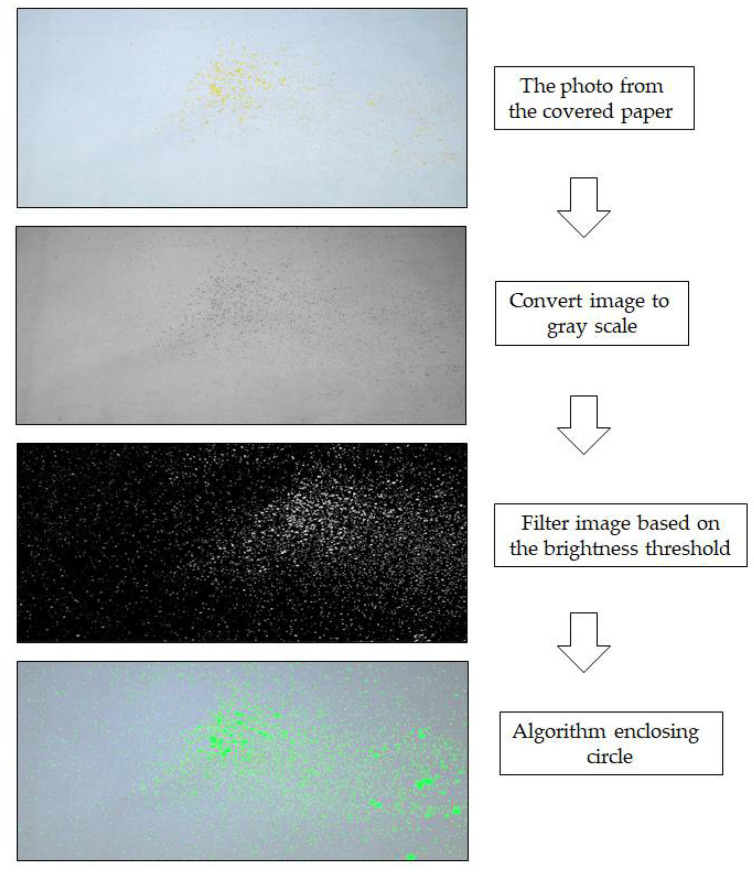
Image processing steps.

The researchers processed the images according to the following steps. First, the covered paper coated with aerosol and splatter particles was placed on the floor until it was completely dry. Second, a digital single-lens reflex camera (Pentax; manual mode setting, focal length = 105 mm, aperture area = F10, and shutter speed = 1/60 s) was used to capture an image of the covered paper. The camera was 210 cm above the ground when it was used to capture the paper. Third, the images were translated to gray scale and identified between the paper and the aerosol and splatter particles. The images were distinguished from the grayscale photos using the brightness threshold point and then converted to black and white. The aerosol and splatter particles were recognized once again using algorithm blob detection [13,14] by analyzing a set of black and white spots in the images and generating circle-like patterns. The algorithm enclosing circle was applied. With the support of this method, a circle was generated by encircling it with spots from the images. As a result, each aerosol and splatter particle was surrounded by a green circle. After, the droplets were surrounded by a green circle. The drops were then a green circle. Finally, the aerosol and splatter particles in the paper were detected and identified. The size of the aerosol and splatter particles was proportionate to the green circle (Figure 3). 

### 2.5. Statistical Analysis

Opencv-python for Python 3.6 was used to create an image processing approach that demonstrated the aerosol and splatter size and count number. The data were analyzed using SPSS version 22.0 (SPSS, Chicago, IL, USA). The differences between the groups were analyzed using the independent *t*-test at the 95% confidence level.

## 3. Results

### 3.1. The Size of the Aerosol and Splatter Particles

The size of the aerosol and splatter particles was measured by the image processing approach. The results are shown in Table 1.

### 3.2. Comparison of the Aerosol and Splatter Particle Size on the Upper and Bottom Board Paper

**Figure 4:**
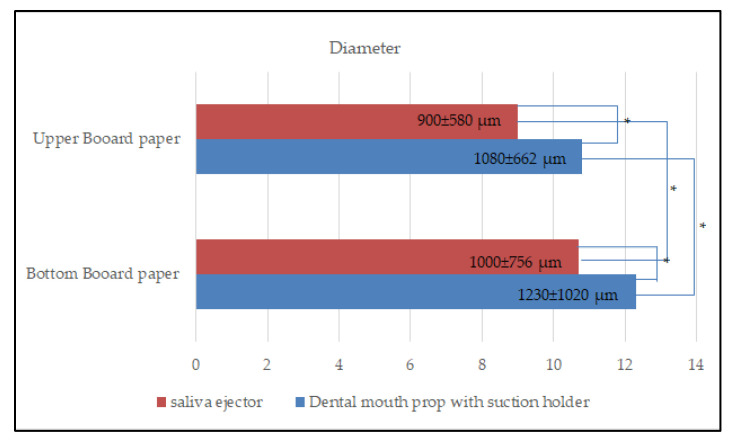
Bar chart showing the difference in the mean diameter of aerosol and splatter particles between upper board paper and bottom board paper when using a saliva ejector and a dental mouth prop with a suction holder. * Statistical significance at *p* < 0.001.

On the upper and bottom board paper, the average diameter of the aerosol and splatter particles was measured. The scaler generated the aerosol particles that were retained on the upper board paper during the use of the dental mouth prop with a suction holder (mean ± SD: 1080 ± 662 µm ranging from 370 to 1307 µm) compared to on the bottom board paper (1230 ± 1020 µm ranging from 370 to 1310 µm). The used saliva ejector diameter of the aerosol size on the upper board paper was 900 ± 580 µm, ranging from 370 to 1260 µm, and on the bottom board paper, it was 1000 ± 756 µm, ranging from 370 to 1300 µm. There was a statistically significant difference (*p* < 0.001) between the two groups, indicating that both dental mouth props with a suction holder and saliva ejector resulted in a larger size on the upper and bottom board papers (Figure 4). Furthermore, the results revealed that there was a statistically significant difference between the two groups on the upper and bottom board papers. Every scaling procedure was completed after being carried out for 5 min. In this study, there was no statistically significant difference between the two device groups in terms of the amount of time spent performing ultrasonic scaling.

### 3.3. Comparison of the Aerosol and Splatter Particle Count Number on the Upper and Bottom Board Paper

**Figure 5:**
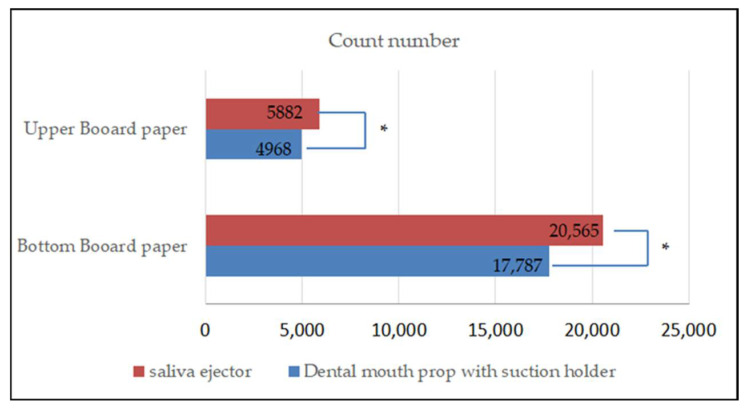
Bar chart showing the difference between the count number of aerosol and splatter particles between the upper board paper and bottom board paper. * Statistical significance at *p* < 0.01.

The results showed that the count number of the aerosol and splatter particles from the use of the dental mouth prop with a suction holder was different from the count number of the saliva ejector (Figure 5). There was a significant difference in the aerosol and splatter count between the two devices (*p* < 0.01).

## 4. Discussion

The generation of aerosol and splatter creates a significant risk for airborne contamination within the dental clinic [1,2,3,4]. Most routine dental treatments are aerosol-generating procedures that produce a mixture of splatter and aerosols that contain saliva, blood, and viable microorganisms (including bacteria and viruses) [1,5]. Commonly used dental instruments and ultrasonic scaling produce the greatest amount of aerosol and splatter, which can be disseminated from the treatment area. Recently, a study investigated a biosafety protocol tool using 3D printing technology called “SUR-FACE” to reduce the aerosol cloud. Nevertheless, the limitation of the technology is its high cost and the patient has to hold it during the procedure [15]. In order to assess the benefit of any suction methods or devices, it is first required to evaluate the size and count number of aerosols and splatter during dental procedures.

Therefore, in this study, comparative dissemination of aerosol and splatter using a suction device during ultrasonic scaling was conducted. The result showed a statistically significant difference in the mean aerosol and splatter diameter between the two suction devices. The particle size was larger when the dental mouth prop with a suction holder was used than when the saliva ejector was sued. Specifically, according to the weight that falls to the ground, the dental mouth prop with a suction holder caused less spreading than the saliva ejector. In addition, when a dental mouth prop with a suction holder was used on both the upper and bottom boards, the average count number of aerosol and splatter particles was less than when a saliva ejector was used. So, it is expected that the spread was reduced because the mean diameter was larger, and the average count number was lesser. 

Aerosol and spatter production during dental scaling in the oral cavity using an ultrasonic scaler has been well documented in the literature [16,17,18,19,20]. This aerosol and spatter might contain infectious agents originating from the patient or the dental unit waterlines that pose a health threat to the dentist, patient, and staff members who are within the spray’s pattern. Due to their heightened susceptibility to the potentially pathogenic microorganisms found in these particles, the impact of these aerosols and splatter on immunocompromised patients is concerning [21,22]. Smaller-sized aerosols are more likely to spread respiratory illnesses. Especially, aerosol particles smaller than 5 μm are more likely to remain airborne for indefinite periods and be deposited in the lower respiratory tract [23]. However, Han et al. [6] found that in splatter and aerosol contamination in dental aerosol-generating procedures, the splatter particle size in the ultrasonic scaler was 281 ± 188 µm, ranging from 200 to 1020 µm. In this study, the splatter particles produced by both devices had an average diameter of 1080 ± 662 and 900 ± 580 µm, which is larger than that found in previous research. The measurement method used in this study may have contributed to the increased diameter of the splatters and aerosols. To construct image processing, the researchers used Opencv-python for Python 3.6. The algorithm was used to build the circle around the particle on the paper after converting the photo from the cover paper to gray scale. The benefits of this technique are that it is simple to use, count the number of particles, measure the diameter, and process. The disadvantages include numerous processing steps and the possibility that the circle will be larger than the realistic size.

According to previous studies, mouth supports make it easier for patients to keep their mouths open during intraoral operations without experiencing extra pain or discomfort. Importantly, research has shown that patients overwhelmingly prefer the use of mouth supports [24,25]. Therefore, researchers developed a dental mouth prop with a suction hole as a soft and gentle mouth prop that attaches to the saliva ejector with a suction hole. The aim of this study was primarily to test the ability of the dental mouth prop with a suction holder device to reduce aerosol and splatter in a simulated clinical study. It had better effects than only the use of the saliva ejector. When working without dental assistance, it can help dentists and dental students to control moisture and produce better work. It is preferable to have less infectious propagation, especially during the COVID-19 period. The dental mouth prop with the suction holder is composed of sterile material that can be sterilized and reused. Furthermore, it may enable patients to maintain an open mouth position for extended periods of time.

However, the low-volume saliva ejector is used to remove water that collects in the floor of the mouth rather than to remove air. Therefore, it may not be a very efficient tool in reducing the aerosol cloud. The combination of a high-volume evacuator with a large bore evacuator tip should be advised for use during ultrasonic scaling. Additionally, there is a chance that airborne contaminants will enter the ventilation system and spread infection. The risk of air contamination can be reduced by the ventilation system’s high-efficiency particulate air (HEPA) filters and UV chambers [2]. Air disinfection with a lamp that produces UV light between 250 and 265 nm has demonstrated extremely high fungicidal, virucidal, and bactericidal action [26]. This is because DNA chains were broken down and proteins are denaturated. However, these techniques are expensive. 

The limitation of this study is that the mouth prop with the suction holder was positioned on the left first molar in this trial. The right side was not tested, so this study did not know how different it was. Furthermore, in the COVID-19 situation [27,28,29], high-volume suction in conjunction with the saliva ejector is recommended; however, this trial did not use this setup, so it is not representative of the real situation. This study only basically tested the device in a simulated clinical study to see how it functioned.

## 5. Conclusions

This study’s results showed that the aerosol and splatter diameters obtained from the use of a dental mouth prop with a suction holder device and a saliva ejector were significantly different (*p* < 0.05).

## Figures and Tables

**Table 1:** The mean ± standard deviation diameter (micrometer) of the aerosol and spatter particle and count number between the two devices.

Device	Time	Upper Board Paper		Bottom Board Paper	
		Diameter (µm)	Count Number	Diameter (µm)	Count Number
Dental mouth prop with suction holder	1	990 ± 668	9055	970 ± 779	16,486
	2	1170 ± 558	2426	1720 ± 1253	13,956
	3	1290 ± 659	3428	1120 ± 910	22,922
	mean	1080 ± 662	4968	1230 ± 1020	17,787
Saliva ejector	1	1340 ± 678	3560	1090 ± 879	24,065
	2	820 ± 486	8836	940 ± 676	19,257
	3	730 ± 503	5253	940 ± 640	18,376
	mean	900 ± 580	5882	1000 ± 756	20,565

## Data Availability

Not applicable.
